# A Survey on IoT Application Architectures

**DOI:** 10.3390/s24165320

**Published:** 2024-08-17

**Authors:** Abdulkadir Dauda, Olivier Flauzac, Florent Nolot

**Affiliations:** LAB-I*, Université de Reims Champagne-Ardenne, 51100 Reims, France; olivier.flauzac@univ-reims.fr (O.F.); florent.nolot@univ-reims.fr (F.N.)

**Keywords:** IoT application, IoT architecture, IoT protocol, cloud, edge, fog

## Abstract

The proliferation of the IoT has led to the development of diverse application architectures to optimize IoT systems’ deployment, operation, and maintenance. This survey provides a comprehensive overview of the existing IoT application architectures, highlighting their key features, strengths, and limitations. The architectures are categorized based on their deployment models, such as cloud, edge, and fog computing approaches, each offering distinct advantages regarding scalability, latency, and resource efficiency. Cloud architectures leverage centralized data processing and storage capabilities to support large-scale IoT applications but often suffer from high latency and bandwidth constraints. Edge architectures mitigate these issues by bringing computation closer to the data source, enhancing real-time processing, and reducing network congestion. Fog architectures combine the strengths of both cloud and edge paradigms, offering a balanced solution for complex IoT environments. This survey also examines emerging trends and technologies in IoT application management, such as the solutions provided by the major IoT service providers like Intel, AWS, Microsoft Azure, and GCP. Through this study, the survey identifies latency, privacy, and deployment difficulties as key areas for future research. It highlights the need to advance IoT Edge architectures to reduce network traffic, improve data privacy, and enhance interoperability by developing multi-application and multi-protocol edge gateways for efficient IoT application management.

## 1. Introduction

The Internet of Things (IoT) has, in recent times, become one of the major enablers for many of the innovations the world has witnessed in many areas of human activities, playing vital roles in key sectors, ranging from manufacturing industries to agriculture, health, education, security, and transportation. For example, IoT devices like sensors and actuators are used in manufacturing to monitor equipment performance, track inventory in real time, optimize supply chains, and enable predictive maintenance [1]. This helps reduce downtime, improve production efficiency, and lower operational costs. The IoT in agriculture, also known as precision agriculture or smart farming, is used to revolutionize traditional farming methods by integrating technology to enhance efficiency, optimize resources, and improve crop yield and quality using devices for soil sensing, crop monitoring, and livestock tracking [2]. Also, in the health sector, it has improved patient monitoring, enabling continuous, remote, and real-time tracking of health parameters using devices such as blood pressure cuffs, glucose meters, and heart rate monitors [3]. This wide area of applicability has made the IoT a complex network, bringing together many heterogeneous devices mostly supported by different protocols and network management tools [4]. To deal with this complexity, numerous architectures, network standards, and protocols have been developed with the intent of making these devices and the systems interoperable [5]. 

In its 2023 publication, Statista showed that the number of IoT-connected devices worldwide has surpassed 15 billion, and this number, based on the growth rate measured from 2019 to 2023, is forecast to almost double by the year 2030 [6]. This avalanche of devices, systems, and technologies used in deploying IoT services underscores the need for an up-to-date classification of the various elements of the IoT systems with a special focus on application architectures, classical protocols used, and deployment platforms.

This survey explored the primary IoT architectures: cloud, edge, and fog architectures. We delved into each architecture, discussing its characteristics, advantages, challenges, and suitability for various applications. We also studied the commonly used IoT protocols and their categorizations. Additionally, in Section 3.2, we highlighted our contribution to edge architecture through our work on a proposed multi-app and multi-protocol IoT Edge gateway for dynamic application management. Detailed information about this architecture can be found in [7]. 

The rest of this paper is organized as follows: Section 2 provides a general overview of IoT architectures; Section 3 discusses reference models/architectures; Section 4 covers IoT protocols and standards; and Section 5 contains the study conclusions. In Section 2 we present an overview of a typical IoT architecture, showing the two connectivity options for IP and non-IP devices with the cloud platforms. In Section 3 we review the classical reference models and architectures for the IoT. This includes models and architectures from standardization organizations, top IT solution providers, and academia to highlight the current state-of-the-art application architecture, focusing on how and where applications and data are managed, processed, and stored. In Section 4 we review the IoT protocols and standards based on the protocol stack presented. In Section 5 we draw conclusions based on our research and findings.

## 2. General Overview of IoT Architectures

The architecture of an IoT involves a complex network of interconnected components that work together to collect, process, analyze, and act upon data generated by IoT devices [8,9]. With an increasing need for IoT solutions that can cope with the wide range of sensor and actuator devices, which are not only heterogeneous in terms of the protocols they use to communicate with other devices but also the data format they are capable of handling, many IoT application architectures have either been proposed or developed. So far, none of the proposed or developed architectures are ideal for all application domains or scenarios [10]. This is due to the interdisciplinary nature of IoT, where each area poses a unique set of requirements. 

IoT architectures usually consist of multiple layers, each with a unique role in the IoT system. These architectures balance centralized control and distributed processing, utilizing cloud, edge, and fog computing paradigms. This optimization of resource utilization, scalability, responsiveness, and reliability meets the diverse requirements of IoT applications across various industries and use cases [11]. Before we delve into the main topics of this survey, we consider the definitions of cloud, edge, and fog computing to comprehend how they are employed to support various IoT application architectures.

According to [12], cloud computing is a computing model that allows users to access various services over the internet. These services include storage, servers, databases, networking, software, analytics, and more. Instead of owning and maintaining their own computing infrastructure or data centers, organizations can rent access to a range of these services from a cloud service provider. 

**Figure 1:**
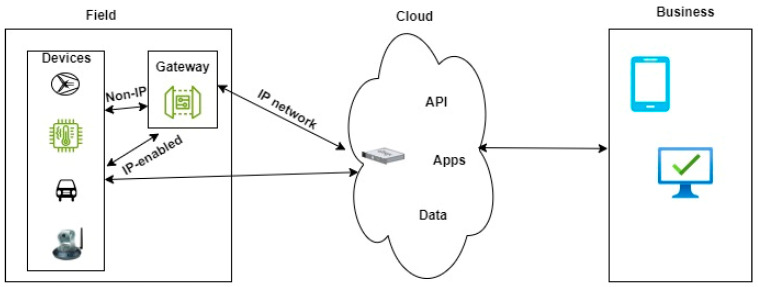
IoT cloud architecture showing device-cloud connectivity options.

In an IoT cloud architecture, devices can connect to cloud platforms through IP-enabled and non-IP-enabled means, depending on the capabilities of the devices and the requirements of the IoT application. These connectivity options are depicted in Figure 1 below.

In a setup where IP-enabled devices are used, they come with built-in networking capabilities. They can communicate directly over the internet using standard IP protocols like TCP/IP and UDP/IP or higher-level protocols like HTTP and REST. On the other hand, non-IP devices lack native networking capabilities, and to establish connectivity with cloud platforms, they require intermediary devices or protocols. In most cases, gateway devices are used as intermediaries between non-IP devices and the cloud platforms. These gateway devices collect data from non-IP devices using proprietary or specialized protocols and transmit them to the cloud over IP-based networks. They also perform protocol translation or conversion to bridge the communication gap between non-IP devices and cloud platforms. Non-IP devices, which are typically lighter than IP devices, may use wireless or wired communication technologies such as the legacy wM-Bus and LoRa to communicate with gateway devices or other intermediary devices within the network [13]. Gateway devices typically use IP-based protocols to communicate with cloud platforms, allowing them to transmit data securely over the internet to cloud services.

For both IP and non-IP devices, cloud platforms such as AWS IoT, Azure IoT, and Google Cloud IoT use edge gateways to facilitate device connectivity, data ingestion, security, and management. These platforms provide support for various communication protocols, device provisioning methods, and authentication mechanisms to enable seamless integration of devices with cloud-based applications and services. While cloud computing offers numerous benefits, it also presents several challenges organizations must address. These challenges include security and privacy, data protection, network latency, and system integration [11]. 

Lately, there has been an increased interest with the ability to conduct computations at the network’s edge, resulting in a computing model known as edge computing. This approach involves bringing computational capabilities closer to the user devices, often at the network’s edge [11]. Instead of relying solely on centralized cloud data centers for processing and storage, edge computing distributes these resources across a network of edge devices, such as gateways and IoT devices. This allows data to be processed and analyzed locally on the edge devices themselves, closer to where it is generated, rather than being transmitted over long distances to centralized data centers. Edge devices often have limited processing power, memory, and energy resources in comparison to cloud servers. This limitation is due to the need for portability, energy efficiency, and cost-effectiveness for deployment in various environments.

Fog computing has emerged as a solution to simplify cloud computing. It is a decentralized computing infrastructure that divides cloud computing into smaller subnetworks and brings the capabilities closer to the edge of the network, closer to the data source or user devices [14]. The objective of fog computing is to provide decentralized processing, storage, and networking services, thereby addressing some of the challenges of cloud computing such as reduced latency, improved security and privacy, and scalability.

Integrating cloud, edge, and fog computing with IoT has significantly enhanced the creation and management of IoT applications. These technologies offer vast computational, network, and storage resources and capabilities, enabling designers to develop customized architectural design approaches that can address a range of requirements and constraints.

## 3. IoT Reference Models/Architectures

**Figure 2:**
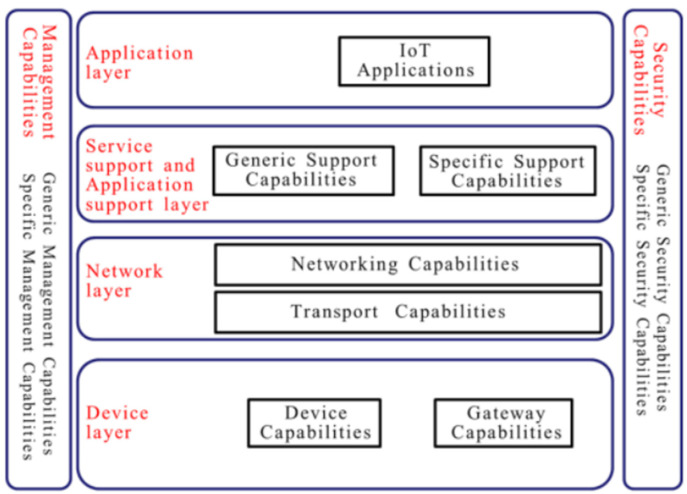
ITU-T Y.2060 IoT Reference Model [15].

The IoT has experienced the development of numerous reference architectures and models by various institutions and consortia. These frameworks provide detailed and comprehensive guidelines for designing, deploying, and managing IoT solutions. The models offer a wide range of solutions to meet the specific needs of different IoT implementations, making it easier for businesses to leverage IoT technology for their operations. The guidelines ensure that IoT solutions are reliable, secure, and efficient, contributing to the success of IoT projects. One example of such a model is the International Telecommunication Union-Telecommunication Standardization Sector (ITU-T) recommendation Y.2060 [15] developed in 2012. This reference model provides a structured approach to conceptualizing the various components and interactions within IoT ecosystems, which is presented in a layered architecture consisting of a device layer, network layer, service support and application support layer, and application layer [15]. This is shown in Figure 2 below.

At its core, the ITU-T Y.2060 Reference Model delineates the key functional domains and their relationships within an IoT environment. These domains encompass the entire IoT lifecycle, from sensing and actuation to data processing, communication, and application services. The layers of the reference model are explained below:

- Device Layer: This is the physical layer where IoT devices are situated. These devices can range from sensors and actuators to industrial machines. They collect data from the environment or perform actions based on instructions received.
- Network Layer: This layer involves the protocols and technologies used to establish connections between IoT devices and the network. It includes Wi-Fi, Bluetooth, Zigbee, cellular networks, etc.
- Service Support and Application Support Layer: This layer acts as a bridge between the device layer and the application layer. It handles data processing, device management, security, and communication between devices and applications. It often includes edge computing resources for processing data closer to the source, reducing latency and bandwidth usage. Protocols used in this layer include MQTT, CoAP, and others.
- Application Layer: This layer consists of the applications or services that utilize the data collected from IoT devices. These applications can range from simple dashboards displaying sensor data to complex analytics platforms such as predictive maintenance systems, AI-driven decision-making engines, or smart city solutions.

The ITU-T has played a significant role in shaping the architectures of the IoT by providing standardization and recommendations for various aspects of IoT deployment. Recently, the focus has shifted to achieving what is known as the new generation IoT (NGIoT), which aims to meet certain architectural properties such as scalability and self-sustainability [15]. These properties are intended to make the solution robust, enabling it to integrate new technologies and operate autonomously for extended periods. This is evident in the recent EU-funded project titled “Architecture for Scalable, Self-*, Human-Centric, Intelligent, Secure, and Tactile Next Generation IoT” (ASSIST-IoT) conceptual reference architecture [16].

The architecture of an IoT application can vary significantly depending on the specific use case, industry, scalability needs, and available resources. It is crucial to design an architecture that strikes a balance between scalability, security, latency, and cost-effectiveness to create a robust and efficient IoT system. According to references [16,17], the principles of scalability, interoperability, security, data management, latency, real-time processing, and autonomy are essential for designing and implementing effective IoT architectures. Each principle tackles specific challenges and requirements, ensuring that IoT systems can meet the demands of various applications.

As IoT continues to evolve, these principles will remain essential in guiding the development of innovative and effective IoT solutions. A well-designed IoT application architecture strikes a balance between these factors while catering to the specific needs of the use case or industry. Flexibility, adaptability, and future-proofing against evolving technology standards and requirements are also integral aspects of a robust IoT application architecture.

While there is no one-size-fits-all architecture, several common paradigms have emerged, each offering distinct advantages and trade-offs. These paradigms are based on the centralized architecture approach where all data processing and decision-making tasks are performed in a central location such as a cloud server or a data center, or in decentralized architecture in which processing tasks are distributed across multiple edge devices or gateways located closer to the data source [18].

Each IoT architecture offers unique benefits and challenges depending on the specific use case, requirements, and constraints. Organizations developing IoT solutions must carefully evaluate these architectures and select the most suitable approach based on factors such as scalability, latency, reliability, security, and cost. Furthermore, ongoing advancements in edge computing, artificial intelligence, and networking technologies have continued to shape and evolve the landscape of IoT architectures, offering new opportunities for innovation and optimization.

### 3.1. IoT Cloud Application Architecture

**Figure 3:**
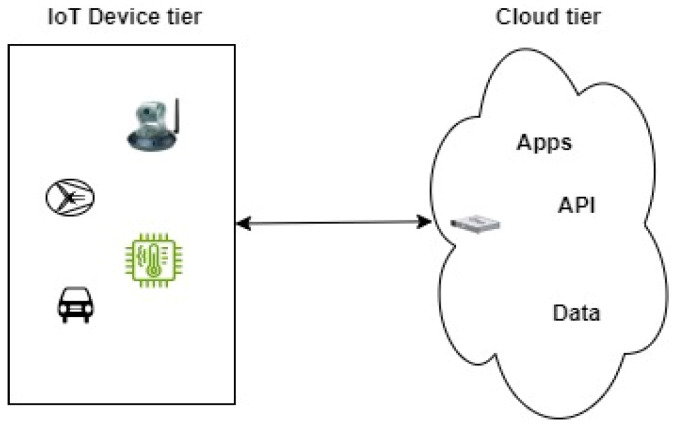
IoT cloud architecture.

At the heart of IoT lies the idea of interconnected devices, spanning from household items to intricate machinery. Such devices produce an enormous amount of data, encompassing everything from sensor data and temperature readings to user inputs and environmental conditions. The true challenge lies not only in obtaining this data but also in leveraging it to make informed decisions. The IoT cloud architectures supply the necessary framework for effortlessly merging IoT devices with cloud-based services, ensuring that data flows seamlessly between the physical and virtual realms [14]. However, its main drawback is the inability of non-IP devices to connect to the cloud platform directly. IoT cloud architecture is depicted in Figure 3 as a one-layered architecture with IP-enabled devices communicating directly to the cloud platform.

**Figure 4:**
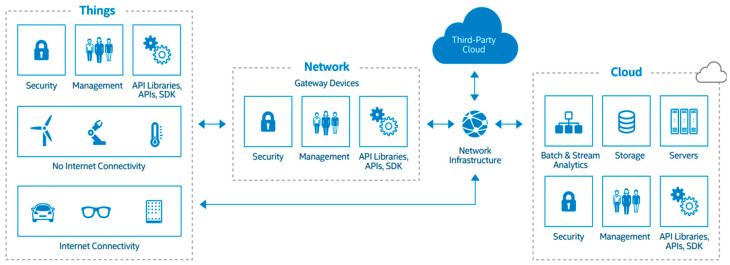
Intel reference architecture for IoT infrastructure [19].

Most of the leading IT and cloud service providers have, over the years, developed solutions for various IoT cloud-based use cases which can be adopted by organizations for their IoT applications. For example, Intel developed a comprehensive reference architecture for building IoT infrastructure, providing a framework for designing scalable, secure, and efficient solutions to address a wide range of IoT applications [19]. Intel’s approach encompassed hardware, software, and services, leveraging the company’s extensive expertise in computing, networking, and data analytics. Specific implementations may vary depending on the use case and requirements. Figure 4 shows components of Intel’s IoT reference architecture.

Intel’s IoT reference model is recommended for a broad range of industries and applications due to its flexibility, scalability, and comprehensive approach to IoT infrastructure. Some key sectors and use cases where Intel’s reference model can be particularly beneficial include industrial IoT (IIoT), smart cities, healthcare, retail, agriculture, energy, and utilities as it offers robust edge computing capabilities and a wide range of connectivity options which enables real-time data processing and analytics. 

Intel distinguishes itself from other leading IoT providers like Amazon Web Services (AWS), Microsoft Azure, and Google Cloud through its unique approach to IoT services. Rather than solely offering cloud platforms for IoT applications, Intel prioritizes providing technologies that extend cloud capabilities to the network’s edge. Additionally, Intel’s collaborative solutions support IoT deployments within cloud environments, bolstering hardware integration, security, and connectivity [19]. 

**Figure 5:**
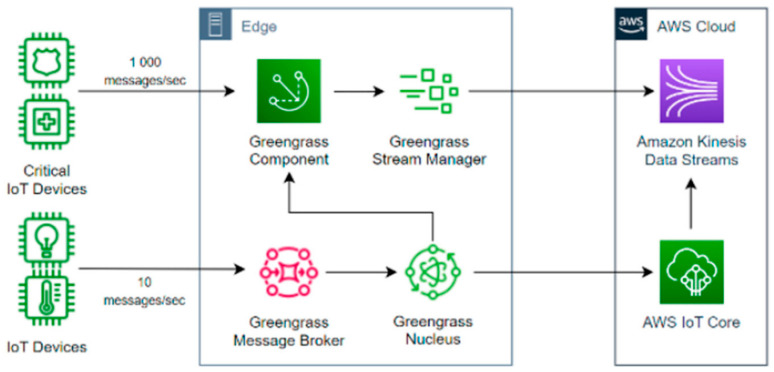
AWS IoT Greengrass stream manager [20].

AWS has developed a variety of hybrid architectures that offer domain-specific solutions tailored to meet the unique requirements of different applications. These solutions, including AWS IoT Greengrass, AWS IoT Fleetwise, and AWS IoT Analytics, cater to the needs of industries such as automotive, agriculture, and healthcare. For instance, AWS IoT Greengrass is an edge computing service that allows devices to collect and analyze data closer to the point of generation, reducing latency and improving responsiveness. It extends AWS functionality to edge devices, enabling local data processing while utilizing the cloud for management, analytics, and storage. Figure 5 illustrates the architecture of AWS IoT Greengrass.

The Greengrass core is the local runtime that resides on edge devices. It enables local execution of AWS Lambda functions, messaging between devices, and secure communications with the cloud. The core component is essential for facilitating local data processing and reducing latency by performing actions directly on the edge device.

**Figure 6:**
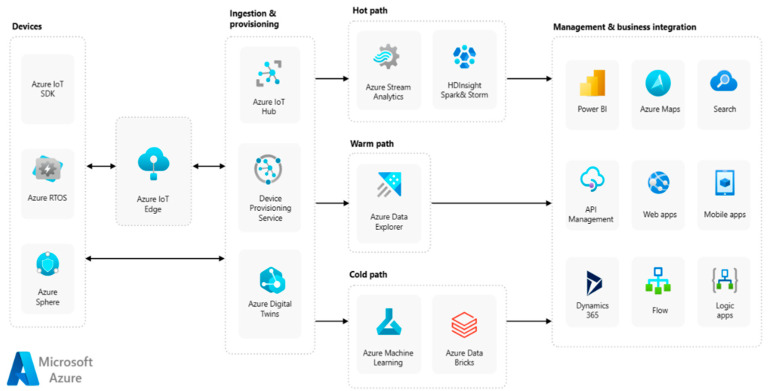
Azure IoT reference architecture [21].

The Microsoft Azure IoT platform offers a flexible and scalable architecture for building IoT solutions that span the entire spectrum from device connectivity to data analytics and application development. Its rich features, seamless integration with Azure services, and strong focus on security and compliance make it a leading choice for organizations looking to harness the power of IoT. The various components of the Azure IoT architecture are shown in Figure 6.

Microsoft Azure IoT provides various services and tools for building end-to-end IoT solutions. It includes Azure IoT Hub for device connectivity, Azure IoT Edge for edge computing, and various analytics and machine learning services for deriving insights from IoT data [21]. 

**Figure 7:**
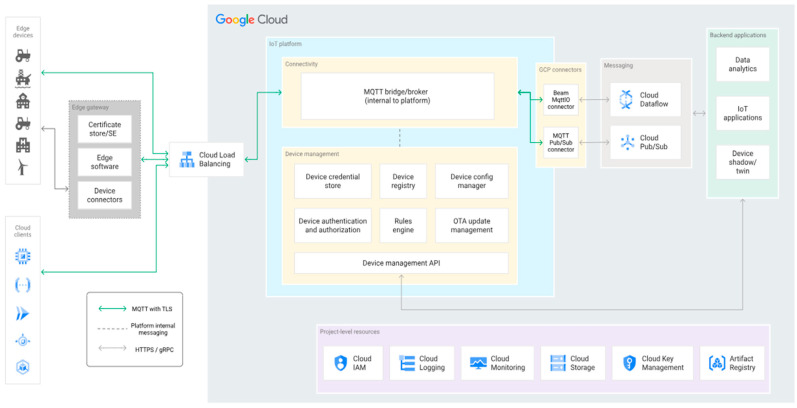
Google Cloud Platform IoT architecture [22].

The Google Cloud Platform (GCP) for IoT is another robust solution for IoT development, deployment, and management at a very large scale. Its suite of services and tools streamlines the process of creating and operating IoT applications while accommodating large-scale deployments with millions of devices [22]. With a focus on high availability, scalability, and reliability, the platform ensures that your IoT applications align with your business needs. The GCP IoT provides an edge computing capability which empowers edge devices and gateways to perform data processing and analysis closer to the data source, making it ideal for low-latency applications or disconnected environments. The various components of the Google Cloud IoT architecture are shown in Figure 7 below.

Apart from the various cloud-based architectures and solutions provided by the technology giants mentioned above, organizations like Capterra [23] provide IoT platforms with full software support for deploying and remotely managing connected devices at any scale. Others, like Eclipse IoT [24], provide open-source solutions for building IoT devices and gateways based on user requirements. 

### 3.2. IoT Edge Application Architecture

In the rapidly evolving landscape of the IoT, where billions of devices are interconnected to collect and exchange vast amounts of data, optimizing the management of IoT applications is paramount. Traditionally, IoT application management has been centralized in cloud servers, as can be seen in [19,20,21,22], reference architectures of some of the major IoT platforms, offering scalability and accessibility but often at the cost of latency, privacy concerns, and dependency on stable internet connectivity. 

Edge computing devices, as mentioned in [25,26], can be used to localize processing and storage. This represents a significant shift towards more efficient, reliable, and secure data management. These benefits of edge computing make it a compelling approach for a wide range of applications, from smart cities and industrial IoT to healthcare and autonomous vehicles. 

**Figure 8:**
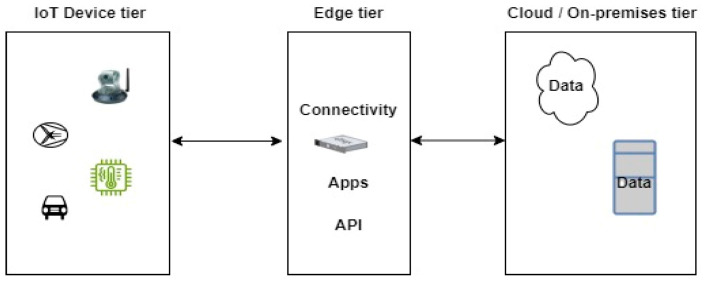
IoT Edge architecture.

Transferring IoT application management from cloud servers to edge gateways is advantageous. This is supported by the work presented in [27,28] on lightweight IoT Edge gateways as services. The diagram of a typical IoT Edge architecture is presented in Figure 8. 

At its core, the transition from cloud server-based to edge gateway-based IoT application management is driven by the need for enhanced performance, privacy, resilience, and cost reduction. Edge gateways, positioned closer to the point of data generation, enable local processing and analysis, thereby reducing latency and enabling real-time decision-making [29,30]. This proximity also addresses privacy concerns by minimizing data transmission over external networks, enhancing data security and compliance with regulatory requirements.

**Figure 9:**
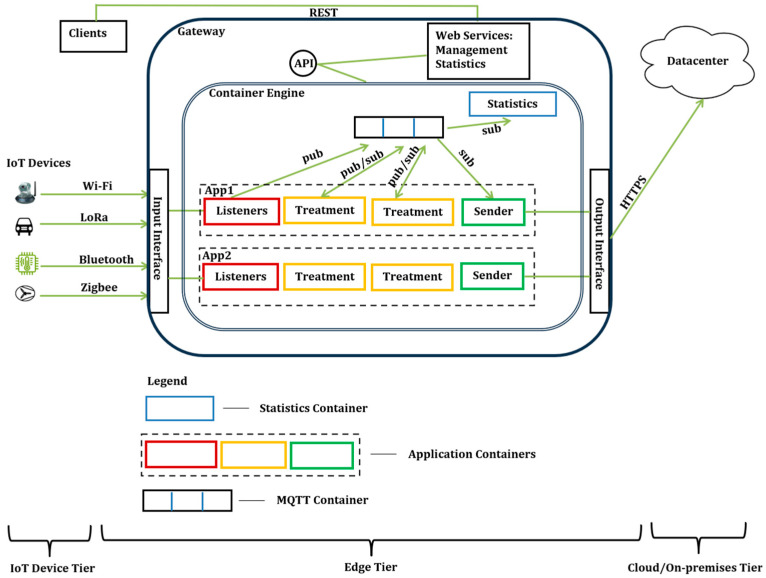
Proposed multi-app multi-protocol edge gateway [7].

Moving from cloud servers to edge gateways poses some difficulties. The main challenge lies in managing heterogeneous devices and ensuring end-to-end data security. This includes carrying out device provisioning, configuration, and orchestration on a constrained edge gateway. To contribute to this, we proposed an IoT Edge gateway architecture that can dynamically handle multiple applications and protocols, as described in our work [7]. The proposed gateway architecture is based on containerized applications designed for single-board computers, such as Raspberry Pi, serving as an edge gateway. Figure 9 shows the gateway architecture.

As shown in Figure 8, the gateway is multi-app and multi-protocol, and data can be sent from the gateway to multiple destinations such as on-premises and cloud data centers. To ensure compatibility, we proposed a JavaScript Object Notation (JSON) model for application management and data exchange within the gateway. This is facilitated using the Message Queuing Telemetry Transport (MQTT) protocol. Application and user management are handled using web services and container management tools. 

The gateway is designed to ensure end-to-end security and safety of execution at different application management levels and during data transfer from the connected devices to the data center. These levels include system, application, and communication levels of security. 

### 3.3. IoT Fog Application Architecture

IoT Fog architecture represents a shift from the traditional cloud-centric model to a more distributed approach. While the cloud remains a powerful resource for storing and analyzing vast amounts of data, it is not always the optimal solution for IoT applications, particularly those requiring real-time responsiveness [11]. 

**Figure 10:**
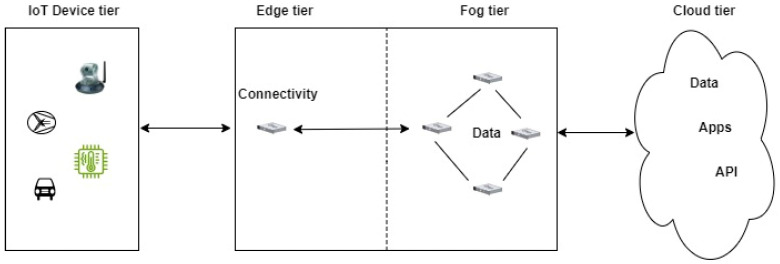
IoT Fog architecture.

Fog computing brings the processing and analysis capabilities closer to the edge of the network, where the data is generated. By deploying fog nodes or edge devices near IoT devices, data can be processed at the point they are generated. This reduces latency and conserves bandwidth since data is filtered and aggregated before being sent to the cloud. The IoT Fog architecture is comprised of three main levels: the edge tier, the fog tier, and the cloud tier. This is shown in Figure 10 below.

Fog architectures are used in distributed-to-centralized data management (D2C-DM) systems to reduce network traffic and latencies, and to improve security levels [31]. The utilization of fog, cloudlet, and cloud technologies was proposed in [32] to manage large volumes of data collected from various sources in a smart city scenario, allowing for more efficient management of urban services and resources.

Also, fog-based synchronization algorithms play a key role in minimizing communication costs and reducing the latency from the cloud to the end devices. An example of such an algorithm is proposed in [33], in which part of the computing and storage work is offloaded to the fog nodes.

## 4. IoT Protocols and Standards

**Figure 11:**
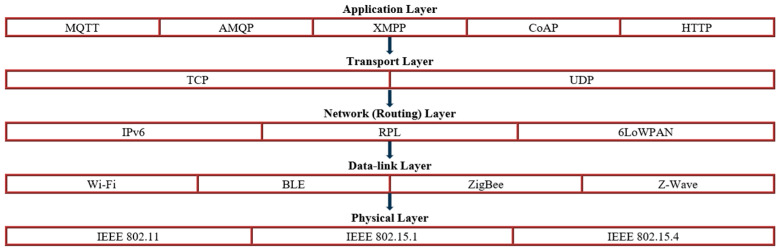
IoT protocol stack; standards IEEE 802.11 [39]; IEEE 802.15.1 [40]; IEEE 802.15.4 [41].

It is crucial to establish IoT protocols and standards to ensure effective, energy-efficient, and secure communication among the various devices and systems in IoT [34]. These devices utilize different protocols across the layers of the OSI (Open Systems Interconnection) model to communicate with each another [35]. Protocols and standards are crucial for determining how devices exchange data and allowing seamless integration across different platforms. IoT protocols are typically categorized into application and network protocols [34,35,36,37,38]. Figure 11 illustrates the IoT protocol stack.

### 4.1. Application Protocols

These protocols govern how data is formatted and communicated between IoT devices and applications. They focus on how data is transmitted, ensuring compatibility and interoperability among various devices and systems. The protocols in this category are based on multiple architectures and communication models, such as publish/subscribe, request/response, and messaging models. Detailed comparisons of various application protocols, including their design goals, security, and supported application types, can be found in [36,37,38,42]. The following are some of the widely used application protocols. They are grouped according to their architectural models.

#### 4.1.1. Queue-Based Publish/Subscribe or Producer/Consumer Models

Queue-based IoT protocols provide unique capabilities to IoT applications with their asynchronous communication, which allows devices to send and receive messages without blocking processes, thereby improving responsiveness and reducing latency. This is particularly beneficial in IoT applications where timely data exchange is crucial, such as in real-time monitoring or control systems.

In this architecture, the publish/subscribe communication model separates the sender (publisher) and the receiver (subscriber), allowing for efficient communication without direct connections. This enables devices to communicate indirectly through a broker, making the system more flexible and scalable. Devices can join or leave the network easily, and new topics can be added without interrupting existing services. The broker architecture in this model can be utilized to provide an improved two-way clock synchronization method for industrial IoT as proposed in [43]. The two most common queue-based protocols are:

- MQTT (Message Queuing Telemetry Transport): A lightweight publish-subscribe messaging protocol ideal for low-bandwidth, high-latency, or unreliable networks. It is commonly used in IoT for efficient communication between devices.
- AMQP (Advanced Message Queuing Protocol): A protocol designed for message-oriented middleware, providing reliable and secure message transmission between devices and applications. In addition to the publish/subscribe model, AMQP supports the producer/consumer model where messages generated by the producers are sent to exchanges for routing to appropriate queues for storage until they are processed by consumers.

#### 4.1.2. Client/Server Models

Client/server protocols operate on a centralized model where client devices communicate with a central server. This architecture simplifies data management and control, as the server acts as the primary repository and processing unit for information. Clients send requests to the server, which processes and responds accordingly, ensuring a streamlined flow of data.

Client/server-based IoT application protocols offer significant architectural benefits, enhancing the efficiency, security, and scalability of IoT systems. Their simplicity, interoperability, and robust security features make them suitable for a wide range of applications, from smart homes to industrial automation. These protocols adhere to widely accepted standards, ensuring compatibility across various devices and platforms. For example, HTTP is universally supported, allowing IoT devices to seamlessly interact with web services, browsers, and other internet-based applications. This interoperability is critical for integrating IoT systems with existing IT infrastructure. CoAP’s multicast support allows a single client request to be sent to multiple servers simultaneously. This is useful for scenarios where the client needs to broadcast a message or command to various devices. Two examples of protocols based on this model are:

- CoAP (Constrained Application Protocol): Designed for constrained IoT devices, CoAP is a protocol that enables communication between devices with limited resources, making it suitable for constrained environments.
- HTTP (Hypertext Transfer Protocol): While primarily known for web browsing, HTTP is also used in IoT for communication between devices and web-based services. It is versatile but may not be suitable for resource-constrained IoT devices due to its overhead.

#### 4.1.3. Messaging Models

- XMPP (Extensible Messaging and Presence Protocol): A versatile communication protocol that has become increasingly important in the context of the IoT. Initially developed for real-time communication, XMPP’s extensibility, security features, and capability to manage presence information makes it a highly suitable option for IoT applications. The protocol, defined by the IETF in RFC 6120 [44] is an open-standard communication protocol based on XML.

XMPP supports both client/server and publish/subscribe communication models. Although primarily designed as a server/client protocol, it also supports a publish/subscribe model through XMPP Extensible Services (XEPs), which extend XMPP’s core services. The PubSub XEP enables this capability [42].

Table 1 below compares these protocols according to their scalability, efficiency, reliability, and security.

### 4.2. Transport Layer Protocols

The two transport layer protocols used in IoT are Transmission Control Protocol (TCP) and User Datagram Protocol (UDP) [45]. TCP and UDP have distinct roles in the IoT landscape, each offering specific advantages while addressing different challenges. TCP’s reliability and data integrity make it ideal for applications where accurate data transmission is critical. In contrast, UDP’s low latency and efficiency are better suited for real-time applications and scenarios where occasional data loss is acceptable [46,47]. Understanding the strengths and limitations of each protocol allows IoT system developers to make informed decisions, optimize communication for various IoT applications, and ensure the effective operation of the interconnected devices.

TCP ensures that application layer protocols receive data accurately and in the correct order. This is crucial for applications like firmware updates, where missing or corrupted data can lead to failures. For example, MQTT, which relies on TCP, benefits from TCP’s ability to handle retransmissions and acknowledgments, ensuring that messages are delivered reliably even in the presence of network issues. UDP supports application layer protocols requiring low latency and minimal overhead, such as real-time sensor data streaming or video transmission. Protocols like CoAP, which operate over UDP, benefit from the reduced latency, making them suitable for time-sensitive IoT applications. UDP does not provide inherent reliability; therefore, application layer protocols incorporate their mechanisms for ensuring data reliability, like retransmissions and acknowledgment messages [46]. Table 1 shows the transport layer protocols’ support for the various application layer protocols and the benefits derived from their features. The performance of different application layer protocols like CoAP and MQTT have been extensively evaluated over TCP and UDP in [48,49] to determine their areas of strength and limitation under different network types and traffic conditions.

### 4.3. Network (Routing) Layer Protocols

IoT networks can be built based on classical network protocols in the communication protocols stack, such as HTTP, TCP/UDP, IPv4/IPv6, and Wi-Fi as defined by IEEE 802.11 [39], or wired networks. However, the classical protocols were not originally intended for low-power and constrained devices as they consume significant resources and energy due to their high operational overhead, making them unsuitable for IoT devices. To address this issue, low-energy versions have been developed to enhance their efficiency. For example, IPv6 over Low-Power Wireless Personal Area Networks (6LoWPAN) has been created to facilitate the operation of IPv6 over the IEEE 802.15 [40] network family, such as Zigbee [5]. The IEEE 802.15.4 standard [41] is the foundation for various low-power, low-data-rate wireless communication protocols. This standard is particularly well-suited for IoT applications, which often require efficient power consumption and sufficient data rates for reliable communication. It defines several data rates, the most common being 250 kbps in the 2.4 GHz frequency band. These rates are adequate for periodic data transmission applications, such as sensor readings and control signals [47,50,51].

Another crucial IoT routing protocol is the IPv6 Routing Protocol for Low-Power and Lossy Networks (RPL) [52]. RPL, standardized by the Internet Engineering Task Force (IETF) in 2011, is specifically tailored for Low-Power and Lossy Networks (LLNs) [53]. These networks are characterized by constrained devices with limited processing power, memory, and energy resources, operating in environments with high packet loss, variable link quality, and frequent topology changes. RPL provides a robust framework for creating and managing such networks, ensuring reliable data delivery while minimizing resource consumption.

### 4.4. Data Link and Physical Layer Protocols

The data link and physical layer protocols are essential for IoT communications. They provide the infrastructure for reliable, efficient, and scalable device interactions. These protocols define how data is transmitted over a network and how devices recognize and communicate with each other on a physical medium. Key protocols in this category include IEEE 802.15.4 [41] for low-rate wireless personal area networks (LR-WPANs), IEEE 802.11 [39] a/b/g/n for Wi-Fi networks, and 2G/3G/LTE for cellular networks [34,54,55].

These protocols offer unique advantages tailored to specific application requirements, such as low power consumption, long-range capabilities, high data rates, and robust error handling. This survey focuses on the capabilities these protocols offer across various application areas. For an in-depth analysis and detailed comparisons, refer to [55,56,57]. See Table 2.

In addition to these protocols, various organizations and consortia like the Institute of Electrical and Electronics Engineers (IEEE), Internet Engineering Task Force (IETF), International Organization for Standardization (ISO), and others develop and maintain standards that encompass aspects such as security, interoperability, and data formats within the IoT space [34]. These standards ensure that devices from different manufacturers can communicate seamlessly, exchange data, and ensure their systems’ security.

## 5. Conclusions

IoT application architectures are continuously evolving to meet the varied and rigorous demands of modern IoT applications. The shift towards hybrid and decentralized models reflects a trend towards more adaptable, resilient, and efficient systems. Emphasis on security, scalability, interoperability, and autonomy continues to drive innovation and advancement in this field. As IoT becomes increasingly integrated into different aspects of life and industry, robust and flexible architectures will be crucial to fully harness the potential of connected technologies and propel future progress. This survey lays the groundwork for comprehending the current landscape and shaping the strategic direction for future IoT architecture development.

## Figures and Tables

**Table 1:** Comparison of application protocols.

Protocol	Message Type	Transport	Scalability	Efficiency	Reliability	Security
MQTT	Publish/ Subscribe	TCP	High, supports many devices	Lightweight, low bandwidth	QoS levels for reliable delivery	Supports TLS/SSL, basic authentication
AMQP	Publish/ Subscribe	TCP	High	Moderate to high	High, supports guaranteed delivery	Supports TLS/SSL
CoAP	Request/ Response	UDP	High, designed for constrained devices	Very low overhead, optimized for low-power devices	Built-in reliability mechanisms	DTLS for encryption
HTTP	Request/ Response	TCP	Moderate	High overhead compared to others	Reliable but lacks inherent message delivery guarantees	Supports HTTPS
XMPP	Publish/ Subscribe and Request/ Response	TCP	Supports on-demand dynamic resource allocation	Designed to minimize overhead	Ensures reliable delivery of messages with receipt acknowledgements	Support TLS/SSL, SASL authentication

**Table 2:** IoT data link and physical layer protocols with application areas [55,56,57].

Protocol	Data Rate	Range	Energy	Application Area
Wi-Fi	High	Broad range	High	Smart home, video streaming, and industrial automation.
Bluetooth Low Energy (BLE)	High	Short	Low	Health monitoring and smart home
Zigbee	Low	Short	Low	Building automation and smart meters
Thread	Low	Short	low	Smart homes and industrial applications
Z-Wave	Low	Short	Low	Home automation systems such as smart locks, thermostats, and lighting controls
Z-Wave Long Range (ZWLR)	Low	Long	Low	Ideal for multi-dwelling units (MDUs)
LoRa (Long Range)	Low	Long	Low	Environmental monitoring, agriculture, and asset tracking

## Data Availability

Not applicable.
